# Healthcare's New Horizon With ChatGPT's Voice and Vision Capabilities: A Leap Beyond Text

**DOI:** 10.7759/cureus.47469

**Published:** 2023-10-22

**Authors:** Reem Temsah, Ibraheem Altamimi, Khalid Alhasan, Mohamad-Hani Temsah, Amr Jamal

**Affiliations:** 1 College of Pharmacy, Alfaisal University, Riyadh, SAU; 2 College of Medicine, King Saud University, Riyadh, SAU; 3 Pediatric Nephrology, King Saud University, Riyadh, SAU; 4 Solid Organ Transplant Center of Excellence, King Faisal Specialist Hospital and Research Centre, Riyadh, SAU; 5 Evidence-Based Health Care & Knowledge Translation Research, King Saud University, Riyadh, SAU

**Keywords:** dalle-3, user-centric interface, image recognition, voice recognition technology, artificial intelligence chatgpt-4

## Abstract

The integration of artificial intelligence (AI) in healthcare is responsible for a paradigm shift in medicine. OpenAI's recent augmentation of their Generative Pre-trained Transformer (ChatGPT) large language model (LLM) with voice and image recognition capabilities (OpenAI, Delaware) presents another potential transformative tool for healthcare. Envision a healthcare setting where professionals engage in dynamic interactions with ChatGPT to navigate the complexities of atypical medical scenarios. In this innovative landscape, practitioners could solicit ChatGPT’s expertise for concise summarizations and insightful extrapolations from a myriad of web-based resources pertaining to similar medical conditions. Furthermore, imagine patients using ChatGPT to identify abnormalities in medical images or skin lesions. While the prospects are diverse, challenges such as suboptimal audio quality and ensuring data security necessitate cautious integration in medical practice. Drawing insights from previous ChatGPT iterations could provide a prudent roadmap for navigating possible challenges. This editorial explores some possible horizons and potential hurdles of ChatGPT's enhanced functionalities in healthcare, emphasizing the importance of continued refinements and vigilance to maximize the benefits while minimizing risks. Through collaborative efforts between AI developers and healthcare professionals, another fusion of AI and healthcare can evolve into enriched patient care and enhanced medical experience.

## Editorial

**Figure 1:**
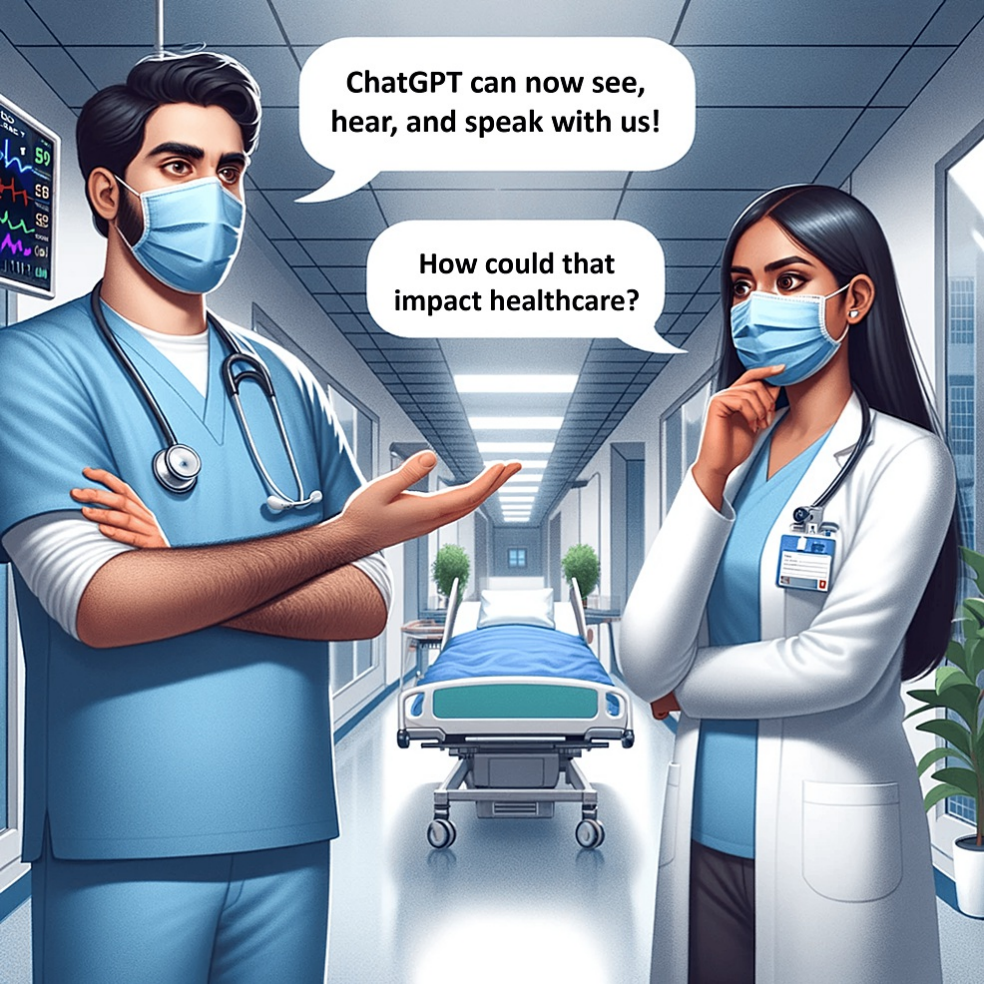
Imaginary example of two healthcare professionals talking about the new features of ChatGPT of voice and image interactions The picture was crafted by the authors on 11 Oct 2023 on ChatGPT-4, using the AI-enabled OpenAI’s DALL·E 3, using the prompt: “Use DALL·E 3 to draw 3D image of 2 healthcare professionals talking together, one saying “ChatGPT can now see, hear and speak with us!” and the other replying: How could that impact healthcare?, digital art”. ChatGPT-4 added to the image the following text: “Prompt: Digital art of a diverse pair of healthcare professionals standing in a well-lit hospital corridor. A professional of Middle Eastern descent in a blue uniform enthusiastically mentions, 'ChatGPT can now see, hear and speak with us!'. The other professional, of Indian descent and wearing a surgical mask hanging from the neck, contemplates and responds, 'How could that impact healthcare?'. Nearby, a hospital bed and vital signs monitor can be seen.” The "DALL·E 3"-generated image was then edited by the authors, to correct the spelling "typo" that DALL·E 3 made. The final image is shown here.

The histories of medicine have often been interspersed by innovative advances in technology. Today, as OpenAI (Delaware, US) expands Generative Pre-trained Transformer's (ChatGPT) new voice and image capabilities, healthcare stands at the crossover of a potentially transformative moment that deserves some reflections from healthcare professionals (Figure 1) [1].

When we asked ChatGPT verbally: “How it can talk with us now”, the answer was “The ability for ChatGPT to "talk" and "hear" is due to advancements in technology and the integration of text-to-speech (TTS) and speech-to-text (STT) systems. As a simple breakdown:

1- Speech-to-text (STT): When you speak, the system captures your voice and converts it into text. This is how I "hear" you.

2- Processing the question: Once your speech is converted to text, I process the text to understand your query and generate a response.

3- Text-to-speech (TTS): After I generate a response in text form, the TTS system converts my text response into speech. This is how I "talk" to you.”

Then, we asked ChatGPT about how it can now “see and interact about images”, and ChatGPT-4 answered:

“My ability to "see" and generate images comes from the integration with DALL·E, another model developed by OpenAI. Here's a brief overview of how it works:

1- Image interpretation: When you provide an image or describe one, DALL·E can generate images based on textual descriptions. It doesn't "see" in the same way humans do, but rather recognizes patterns and structures in the data to produce relevant images.

2- Image generation: If you provide a textual description without an actual image, DALL·E can create a new image from scratch based on that description. This is achieved through a vast amount of training data where the model has learned various visual concepts.

3- Interaction: When you ask questions or make comments about the images, I combine the capabilities of ChatGPT with the image-generation abilities of DALL·E to respond and provide context, further descriptions, or generate new images based on your feedback.

It's essential to note that while I (ChatGPT) can generate and interpret images, I don't have consciousness or emotions. I rely on patterns and the data I was trained on to provide responses and generate visuals.”

The promise of progress

From the outset, large language models (LLMs), like ChatGPT, have shown unprecedented promise in revolutionizing healthcare. Their rapid development speaks volumes about their potential to bridge the gap between healthcare workers (HCWs) and patients [2]. With OpenAI's recent enhancements, this potential has multiplied manifold. In Table 1, we suggest hypothetical examples and potential challenges, keeping in mind that users should always approach the technology responsibly, keeping its limitations in mind. As a rule, new technologies are tools designed to assist humans, and should never replace professional medical judgment [2].

**Table 1:** Reflections on ChatGPT's New “See, Hear, and Speak” Features, Possible Healthcare Applications, and Potential Challenges HCWs: Healthcare Workers; ChatGPT: Chat Generative Pre-trained Transformer

Feature	Description	Examples in Healthcare	Potential Challenges
Image Interaction	Users can show one or more images to ChatGPT for analysis and discussion.	- Patients can share pictures of their wounds, rashes, or other visible medical issues for preliminary assessment. - HCWs can quickly share medical images, graphs, or charts for instant analysis and second opinions.	- Complex medical images may be misinterpreted. - Security and privacy of sensitive medical images need to be ensured.
Drawing Tool	Users can guide the assistant’s attention to specific parts of the image.	- Highlighting specific areas of concern on a medical image, like a particular spot on an X-ray or MRI. - Drawing attention to specific symptoms or issues in pictures shared by patients.	- Users might not correctly highlight the areas, leading to misinterpretation. - The drawing tool's accuracy and ease of use are crucial for effective communication.
Multimodal GPT-3.5 & GPT-4	Powers the understanding of images, enabling reasoning and analysis based on the content.	- Interpretation of various medical images and photographs. - Analyzing and providing insights on screenshots of medical records or lab results.	- Limitations in understanding complex or ambiguous medical images. - Ensuring the model's interpretations are accurate and reliable is crucial.
Voice Interaction	Users can engage in voice conversations with ChatGPT, providing a more intuitive interaction mode.	- Patients can verbally describe their symptoms or concerns. - HCWs can verbally communicate complex medical information, making the interaction seamless.	- Suboptimal audio may lead to miscommunication. - Security of voice data and prevention of impersonation or fraud is necessary.
Voice Translation Feature	Facilitates translation into various languages in the user's voice.	- Assisting in communication between HCWs and patients who speak different languages. - Possible future application in translating sign language.	- Accuracy of translation in medical terminology is vital. - It may not be proficient in all languages or dialects, leading to potential misunderstandings.
"Vision" Usefulness	Vision is designed to be a useful tool while respecting users' privacy is vital.	- Can assist blind or low-vision patients in understanding visual information in their surroundings.	- The system should not inaccurately analyze or make misleading statements about images. - Privacy concerns regarding individuals appearing in the background of images need to be addressed.
Model Limitations Transparency	OpenAI is transparent about the model's limitations and advises against high-risk use without verification.	- Users are informed about potential limitations, promoting responsible use in healthcare scenarios.	- Users must be cautious while relying on the model for medical topics and should always seek professional verification.
Expanded Access	The new capabilities will be available to Plus and Enterprise users, with possible plans for broader access in the future.	- Broader access will enable a larger group of HCWs and patients to utilize these capabilities for healthcare purposes.	- With expanded access, managing and monitoring the "responsible" use of the technology becomes imperative.

The new voice and image interface is innately user-friendly. Be it a healthcare professional wanting a second opinion on unfamiliar medical images or patients seeking clarifications on a prescription, interacting with ChatGPT could become insightful. The capability to snap an image, whether it's a medical report or a skin lesion, and converse about it, breaks down previously existent barriers. Voice interaction further smoothens this experience, creating a more human-like conversation [3].

Additionally, in our globalized world, language can sometimes be a barrier. ChatGPT and other LLMs’ voice capabilities could be game changers in breaking down these linguistic walls, aiding in the translation between patients and HCWs, and potentially even interpreting sign language in the future [3].

Obstacles ahead

While the horizon could be promising, it is essential to proceed with caution. Voice communication is susceptible to suboptimal audio, which can be detrimental in critical healthcare scenarios. Miscommunication, either due to inaccurate image processing or voice interpretation, remains a challenge, especially with intricate medical images [4].

Furthermore, the security of patients' voice samples and images is paramount. It is crucial to ensure optimized patient and healthcare systems identity security and confidentiality, in an era where cyber security and data breaches are central [5].

Learning from the past

While we embrace new LLM capabilities, it is essential to remember the lessons from the “older” models. Previous limitations of using ChatGPT in healthcare, such as accuracy or ethical concerns, may mirror themselves in some of the advanced models. As a result, heightened vigilance is required, along with accelerated research efforts to address these aspects, ensuring that our understanding evolves in tandem with the rapidly progressing LLMs.

Conclusions

While OpenAI's advanced voice, vision, and text capabilities within ChatGPT provide a stride in AI tools available to healthcare professionals and patients, the adoption of such tools is not without new challenges. On the positive side, these innovations can offer streamlined diagnostics support, facilitate patient education, and promote real-time access to vast amounts of information in everyday medical practice. However, they also come with intrinsic limitations: AI interpretations are only as robust as the data they are trained on, and the risk of bias or inaccuracies persists. For healthcare professionals, it is imperative to critically appraise and judiciously utilize these tools, avoiding over-reliance and recognizing AI's current limitations. Future researchers, from both healthcare and AI experts, have the responsibility to enhance these models and investigate their short- and long-term consequences, ethical considerations, and possible challenges in clinical contexts. Thorough assessments and continuous feedback mechanisms are crucial to realize the potential of AI in healthcare, ensuring patient welfare and the quality of care are maintained.
